# 

**Published:** 1886-02

**Authors:** 


10 cts. a copy. **FEB., 1886.** $1.00 per year.

### HALL'S JOURNAL of HEALTH

ESTABLISHED 1854

**OFFICE, 75 & 77 BARCLAY STREET, NEW YORK.**

Entered as Second-class Matter at the Post-Office, New York.

